# A Comparative Study on the Use of Smartphone Cameras in Photogrammetry Applications

**DOI:** 10.3390/s24227311

**Published:** 2024-11-15

**Authors:** Photis Patonis

**Affiliations:** School of Rural & Surveying Engineering, Aristotle University of Thessaloniki, University Box 439, GR-54 124 Thessaloniki, Greece; patonis@auth.gr

**Keywords:** smartphones, close-range photogrammetry, distortions, accuracy

## Abstract

The evaluation of smartphone camera technology for close-range photogrammetry includes assessing captured photos for 3D measurement. In this work, experiments are conducted on many smartphones to study distortion levels and accuracy performance in close-range photogrammetry applications. Analytical methods and specialized digital tools are employed to evaluate the results. OpenCV functions estimate the distortions introduced by the lens. Diagrams, evaluation images, statistical quantities, and indicators are utilized to compare the results among sensors. The accuracy achieved in photogrammetry is examined using the photogrammetric bundle adjustment in a real-world application. In the end, generalized conclusions are drawn regarding this technology’s use in close-range photogrammetry applications.

## 1. Introduction

The evolution of smartphone camera technology changed how digital images are captured and shared. Smartphones are widely available [1], and the use of advanced technology in their cameras has transformed photography from an advanced skill in the past to an easy-to-use experience today [2]. These systems have enhanced capabilities like digital zoom, scene recognition, fully automatic adjustments based on lighting, and image stabilization. The latest smartphones have image sensors with resolutions exceeding 64 megapixels, featuring multiple cameras with various focal lengths, wide-angle cameras, telephoto lenses, ultra-wide lenses, and depth sensors [3]. In addition, built-in GNSS, inertial, and magnetic sensors [4] enable them to be utilized in advanced geomatic applications [5,6]. The disadvantages of smartphone cameras include reduced image quality and increased noise due to smaller pixels [7]. Smartphones’ main cameras have tiny lenses, typically fixed, wide-angle, and with no optical zoom. Furthermore, smartphone cameras may have difficulty with low-light conditions, high-dynamic-range scenes, and lens distortion. Therefore, the quality of photos is not comparable to that of those taken with a high-quality glass lens of a good still camera.

Photogrammetry is the science of making three-dimensional measurements from photographs, mainly used to create 3D models from 2D images. Photogrammetry has numerous advantages over other surveying methods, such as its accuracy, low cost, and speed. It can be used in various applications, such as topographic mapping, monument documentation, civil engineering, etc. [8,9,10,11].

Smartphone cameras can become advantageous tools for photogrammetry due to their accessibility, portability, and improved image quality. In the literature, there are few studies of smartphones in photogrammetry applications, including an evaluation of the results. In the paper [12], the geometric stability of fourteen smartphone cameras was evaluated. Smartphones were calibrated on a checkerboard test field while repeating the process several times. It was found that most smartphone cameras have a lower stability of the internal orientation parameters than a Digital Single-Lens Reflex camera. The study concluded there is real potential in smartphone photogrammetry, but it also has its limits. In a case study [13] comparing a smartphone camera and an affordable full-frame mirrorless camera, it was found that in fair lighting conditions and at a close range, the smartphone camera is comparable to the still camera in terms of image quality. However, a lag was observed in the accuracy of the photogrammetric processes, attributed to the superior quality of the camera lens, which introduces fewer distortions into the images. In the study [14], three models of smartphones were tested to assess if the quality of the topographic reconstruction varies from one model to another. Centimetric (cm) accuracy in Structure-from-Motion point clouds was achieved, regardless of the smartphone model used and when mixing photographs from different smartphones. The results obtained were similar to those obtained by a Terrestrial Laser Scanner.

This paper compares smartphone camera performance in terms of distortion levels and accuracy for close-range photogrammetry applications. Its experimental findings will contribute to research on the feasibility of using this technology in photogrammetry and discuss applications, benefits, limitations, and best practices.

Regarding the structure of the work, after this introductory section, the methods used to evaluate and compare the cameras are discussed. Next, the experimental results for the cameras’ evaluation and a comparison of distortion levels and accuracy performance are presented. This is followed by a discussion on the analysis and interpretation of the results. In the end, the most important conclusions are drawn from the current study.

## 2. Materials and Methods

Ten smartphone devices are used to evaluate camera technology and its potential for photogrammetry. Some are the same model but different devices. However, it was decided to participate in the experiment to determine if the results would be repeated or altered depending on the device. Table 1 shows the technical characteristics of all the devices utilized in the experiments. The technical specifications for the cameras of the smartphones were taken from [15,16].

The evaluation is divided into three sections. The first section focuses on analyzing the distortions introduced into the images. The second focuses on the accuracy of calculating actual coordinates using a purely photogrammetric method. The third section examines the relationship between distortion, accuracy, and cameras’ technical characteristics.

Tools and methods developed and implemented for this purpose are used for the evaluation. Specifically, the software Surveyor-Photogrammetry Version 6.1 is used, which was presented in the paper [17] and used in the study [13]. The software is a collection of tools that are used in education and research. It is written in the Java programming language and uses the JavaFX, Jama, Apache common math, and OpenCV libraries. Development began in 2019 and has been continuously updated.

The distortions in the digital images caused by the lenses are calculated using a calibration method proposed by Zhang [18]. This method is implemented in the OpenCV library [19] and can be used in Java as the function *Calib3d.calibrateCamera* [20]. The camera calibration is based on photographing a checkerboard, where the image and object coordinates of the square’s corner points are known. In this case, a checkerboard with 1813 (49 × 37) control points was used. A sample set of five photographs taken from specific angles was used, as suggested as a standard in [17]. The results of the calibration are the intrinsic matrix and the matrix of distortion coefficients.

Considering that the image sensors of smartphones have different numbers and sizes of pixels, there are two ways to accurately compare their distortions. One is with normalized pixels, where the distortion is divided by a commonly defined sensor dimension, and the other is by converting the distortions into real-world units of measurement—for example, micrometers (μm). The most accurate method is to convert the measurement units into real units, but this requires the pixel size for each device. Otherwise, the device must be excluded from the experiment. In this way, the distortions can be plotted in a single distribution diagram in which their quantitative concentrations and ranges of variation are determined. Each pixel of the digital image is then colored with a color gradient proportional to the distortion. In this way, it is possible to visualize the distortions over the entire surface of the imaging sensor for each camera.

**Figure 1:**
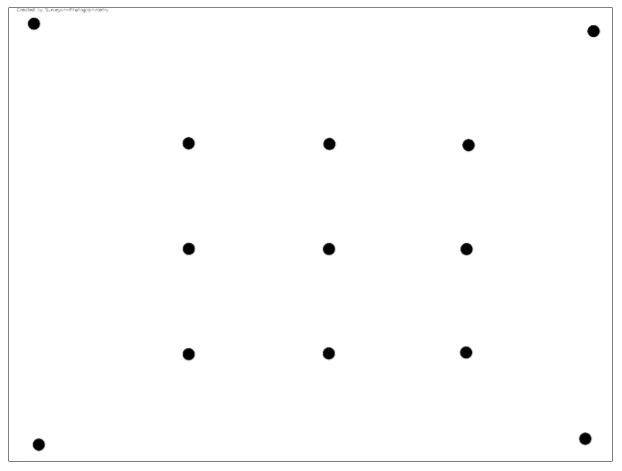
The layout of the control points used for the rectification procedure.

The performance of the image undistortion is then evaluated. Each image is corrected for distortion using the OpenCV function *Calib3d.undistort* [20], where the inputs are the original image, the intrinsic matrix, and the matrix of distortion coefficients. The function returns the undistorted image. The single-image correction method [21] is applied separately to the original and undistorted image, using the central projection to correct the images. The coordinates of the control points of the rectified images can be compared with their actual values to calculate the accuracy achieved for each control point. These results can be displayed in digital images with the same dimensions as the original and appropriate color gradations corresponding to the different accuracy intervals. The comparison between the original and the undistorted rectified image yields the performance of applying the distortion coefficients for image undistortion. This comparison is displayed in an evaluation image with color gradients for the improvement or deterioration of the performance. From this process, the “Rect” indicator is calculated, which expresses the overall percentage improvement or degradation of the image when it is undistorted using the distortion coefficients. The overall methodology is presented in detail in [17], the only difference being that the rectification process now uses thirteen control points (see Figure 1) instead of five in the previous version of the software to improve the control of the process.

The photogrammetric bundle adjustment method is employed to evaluate the accuracy of the calculation of the actual coordinates. A test field is used so that the results and conclusions drawn represent real-world applications of close-range photogrammetry. The process is carried out twice. During the second time, lens distortion is considered. By comparing the results of both solutions, it becomes clear what net effect distortions have on calculating the actual coordinates. To check accuracy, control points whose coordinates have been measured and calculated with high accuracy using a topographic method are used. The differences in the coordinate values between the photogrammetric and topographic methods are expressed by the Root Mean Square Error (RMSE). The synthesis of the RMSE values of all cameras will estimate the accuracy this technology can achieve in the photogrammetric calculation of the actual coordinates.

The correlation between the accuracy of calculating actual coordinates, the calibration’s evaluation parameters, and the sensors’ technical characteristics is examined. First, the normalized values of the parameters are presented in a single diagram to compare them. Scatter charts examine whether the data of the selected parameters are correlated in linear deviation, as determined by linear regression.

## 3. Camera Evaluation

The camera evaluation is divided into three sections. The first section deals with the study of distortions and the evaluation of the undistortion procedure. The second section focuses on the accuracy achieved when calculating the checkpoint coordinates using the photogrammetric bundle adjustment method with additional parameters [22]. The procedure is repeated twice. During the second time, in addition to the checkpoint coordinates and internal orientation, the distortion coefficients are also considered unknowns. In this way, the actual impact of these coefficients on the accuracy achieved for a real-size subject is evaluated. Alternatively, the undistorted images from the initial evaluation section could be used, but this is not the standard workflow for processing these data in photogrammetry. Finally, in the third section, the information from the previous two sections is correlated to determine the relationship between the mean accuracy in the calculation of the coordinates, the mean distortion, the pixel size, the sensor area, and the total number of pixels.

### 3.1. Study of the Distortion Caused by the Camera Lens

**Figure 2:**
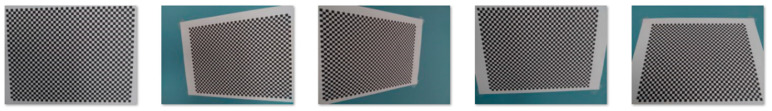
A set of photographs showing the checkerboard pattern used for camera calibration.

To study the distortions, a control field must be photographed. In this method, a checkerboard pattern was used for this purpose. Figure 2 shows an example of a set of five photographs showing the checkerboard used for camera calibration.

The calibration procedure is carried out in Surveyor-Photogrammetry version 6.1. The input is the five photos of the checkerboard, the size of the checkerboard square, the pixel size of the image sensor, and the horizontal and vertical control points shown in the checkerboard. The output is a calibration report, with all the information provided by the OpenCV function that performs the camera calibration. Among other results, the function returns the intrinsic matrix and the distortion coefficient matrix. The accuracy of the calibration is described by the total re-projection error. The smaller this error is, the better the results. The re-projection errors for all camera calibrations are listed in Table 2.

The intrinsic matrix and the matrix of distortion coefficients, as they result from the calibration, are shown in Table 3 and Table 4. The intrinsic matrix lists the focal length (*f_x_*, *f_y_*), the coordinates of the primary point in relation to the upper left corner of the image (*c_x_*, *c_y_*), the estimate of the aspect ratio *AspectRatio* (*f_y_*/*f_x_*), and the calibrated focal length c in millimeters (mm). The values of the parameters *k*_1_, *k*_2_, *k*_3_, *p*_1_, and *p*_2_ are presented in the table of distortion coefficients.

**Figure 3:**
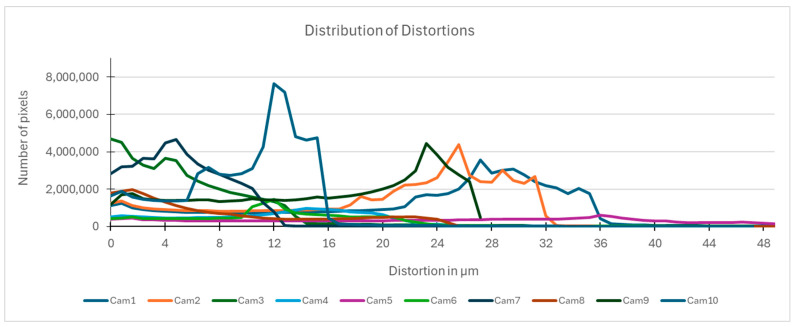
Distribution of Distortions for all smartphone cameras.

By determining the distortion for each pixel using the parameters of the distortion coefficients and the mathematical model used to describe them, it is possible to calculate a distortion distribution for each smartphone camera. The results are plotted in a single diagram (see Figure 3) to allow for comparison. This graph’s primary objective is to determine the range where the distortion values lie. This helps define the color gradations in micrometers that graphically represent the distortions in the evaluation images. The plot is initially calculated using the integer values of the pixels so that the distortion values can be accumulated to discrete values. The pixels are then converted to micrometers based on their actual size. This way, all camera data can be observed in a common diagram. In some cases, this approach approximates the chart’s values, but this does not affect its purpose.

**Figure 4:**
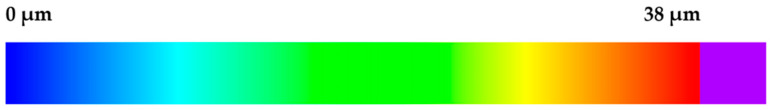
Distortions color gradients to micrometers.

Figure 3 shows that the volume of the distortion values ranges from 0 µm to 38 µm. Pixels with distortion values outside this range are at the edges of the images, where the mathematical model has difficulty describing the distortion due to a lack of control points. This means that values above this threshold can be grouped into one color without any issues. By setting the upper limit of the main volume of the distortions, the color gradations available for their more detailed representation are extended. Figure 4 shows the ranking of the distortion values in relation to the color gradations. The distortion value 0 μm is assigned to the blue color, and 38 μm is assigned to the red. The distortion values in between are assigned proportionally to the color gradients. Values exceeding this limit are evenly represented by the purple color.

Using the color gradients of Figure 4, evaluation images are created where the distortions are now spatially displayed on the surface of the images; see Table 5. In each table entry, the mean distortion is listed along with its standard deviation in pixels and micrometers.

Calculating the distortion for each pixel of the digital image is computationally expensive. For this purpose, concurrent programming was employed, accelerating the process and the application’s functionality. This technique assigns chunks of calculations to different threads, using all processor cores, accelerating the calculation times by approximately half.

To evaluate the performance of the distortion coefficients, the maximum rectification error is calculated for the distorted and undistorted images for all cameras; see Table 6.

**Figure 5:**
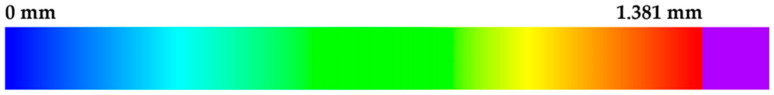
Rectification Error color gradients to millimeters.

The purpose of the maximum rectification errors and Table 6 is to select the threshold value of the rectification error, beyond which the extreme values of the errors, normally located at the edges of the digital images, are grouped and displayed with a single color. In this way, the maximum range of color variations is available for the topological description of most errors in the central areas of the images, which are of greatest importance in photogrammetry. As can be seen in Table 6, in almost all cases, the largest values of the rectification error are found in the distorted image column. From this column, the value of 1.381 mm is selected from the largest observed values of 1.381, 1.728, and 1.977 mm, which is regarded as characteristic of the delimitation of the extreme error values. From the value 0 to the threshold selected, the color values are graded from blue to red. Values that exceed this limit are shown in purple; see Figure 5.

The differences between the rectification of the distorted and undistorted evaluation image are also represented by color gradients, which have distinct values; see Table 7.

The visualized results of the accuracy achieved in the rectification of the distorted and undistorted versions of the evaluation images, as well as their differences which represent the undistortion performance, are displayed in Table 8.

The first column of Table 8 shows the accuracy achieved in photogrammetric single-image rectification when the original image is used. The second column shows the corresponding result when the distortion-corrected image coordinates are used. The third column shows the difference between the two images, which indicates an improvement or deterioration in accuracy depending on the color.

The mean error values with their standard deviations in the rectification procedure of the distorted and undistorted evaluation images and the ‘Rect’ indicator are included in Table 9.

Table 9 shows the performance of the undistortion procedure. The column with the ‘Rect’ indicator is of primary interest. As a percentage, this indicator expresses the overall improvement or worsening of the results. Therefore, it evaluates the quality of the distortion coefficients and, thus, the calibration process. Together with the evaluation images of Table 8, the above indicator can quantitatively and topologically describe the correction performance of checkpoints.

### 3.2. Study of the Accuracy Achieved in a Photogrammetry Application

**Figure 6:**
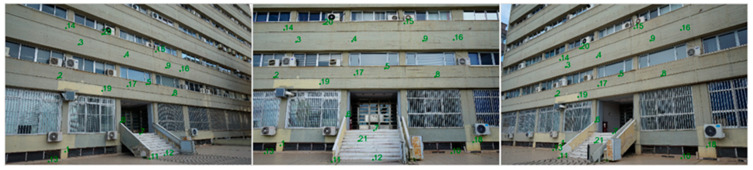
The converged photos of the control field.

To examine the accuracy of calculating point coordinates, a full-scale subject had to be photogrammetrically surveyed. In this way, any results and conclusions obtained will be realistic for close-range photogrammetry applications. The facade of a building was chosen as the subject and three convergent photographs were taken by each camera. The average shooting distance was eleven meters. Figure 6 shows an example of three photographs depicting the area of interest.

**Figure 7:**
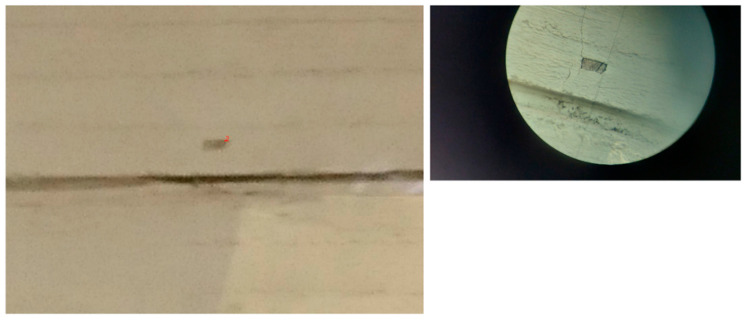
A control point in the building’s facade. **Left**: In the actual photo. **Right**: Through the total station’s telescope.

Points with known coordinates must be available for photogrammetry and to evaluate the accuracy of the coordinate calculation. These Ground Control Points (GCPs), or control points, must be well defined and visible in the photographs. The GCPs include twenty-one characteristic point details of the building façade; see Figure 7 as an example. Ten were employed as control points, and eleven were employed as checkpoints.

The GCPs were measured with a total station in reflectorless mode from the same station and their coordinates were calculated using topographic methods [23], with an estimated accuracy of 2 mm.

**Figure 8:**
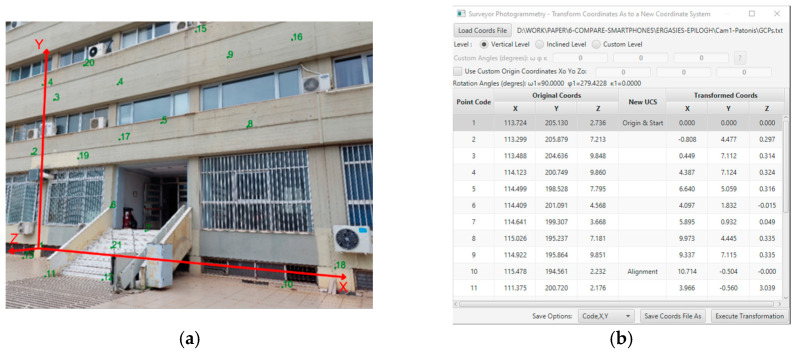
The transformation of the Control Point Coordinates to a New Reference System. (**a**) The new Reference Coordinate System. (**b**) The coordinates transformation on the Surveyor-Photogrammetry software version 6.1.

The coordinate reference system was defined to have the X-axis parallel to the building facade, the Y-axis vertical to the ground, and the Z-axis perpendicular to the XY plane; see Figure 8a. The coordinates transformation was performed using the Surveyor-Photogrammetry software version 6.1; see Figure 8b.

**Figure 9:**
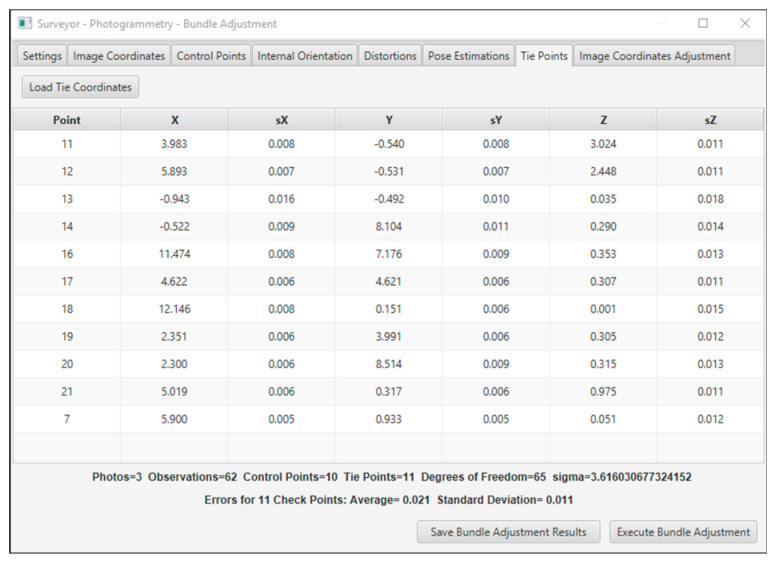
An instance of the bundle adjustment function on the Surveyor-Photogrammetry software version 6.1.

The bundle adjustment with additional parameters was used to calculate checkpoint coordinates. The method was performed twice for each smartphone. Once, the focal length and coordinates of the primary point were included as unknown parameters, and in the second, the distortion coefficients were added to the first case’s additional parameters. The bundle adjustment solution was performed in the Surveyor-Photogrammetry software version 6.1; see an instance in Figure 9. The method accepts the image and the ground coordinates of the control points as the input and estimates the coordinates of the checkpoints.

Table 10 shows the basic elements of the two solutions, such as the number of observations, the checkpoints, the bundle adjustment’s sigma, and the degrees of freedom.

The RMSEs between the photogrammetrically calculated checkpoints and those calculated by the topographic method, for each axis, are listed in Table 11.

In addition, the RMSE, in the XY plane, and the three-dimensional space (3D) are also calculated and are presented in Table 12.

To generalize the conclusions of the evaluation regarding the accuracy in calculating the coordinates, it is necessary to estimate quantities that express the overall accuracy of the examined parameters. For this reason, the RMSE means are calculated for the X, Y, and Z axes as well as for the XY plane and 3D, considering the RMSE errors of all cameras involved in the experiment by using data from Table 11 and Table 12. The estimate of the maximum error that can occur with a probability of 95.44% [24] for the distorted and undistorted cases is also listed in Table 13. The mean RMSE is an empirical statistical quantity but provides a general estimate of the accuracy this technology can achieve.

### 3.3. Study of the Correlation Between the Accuracy and Camera Parameters

**Figure 10:**
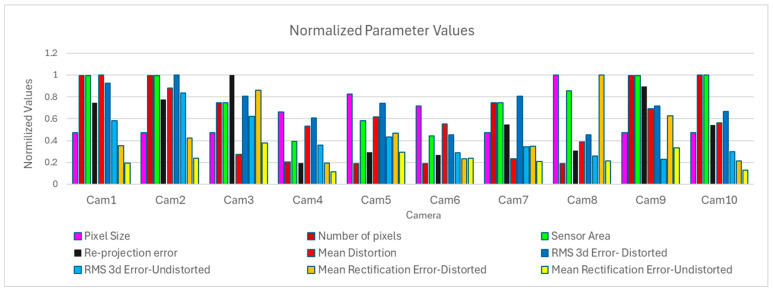
Normalized values of the Pixel size, Number of pixels, Sensor Area, RMS 3D error, Mean Rectification error, Re-projection error, and Mean Distortion.

To relate the accuracy in calculating the actual coordinates to the distortion, the pixel size, the number of pixels, the sensor area, the error in rectifying the images, and the re-projection error, the diagram in Figure 10 was created. The diagram shows the normalized values of the parameters, i.e., the maximum value is always equal to one. This makes it easier to relate the parameters, as they have different units. The 3D error was chosen to represent accuracy, as it includes all coordinates. The diagram shows the RMSE and the rectification error for distorted and undistorted image coordinates for each camera.

**Figure 11:**
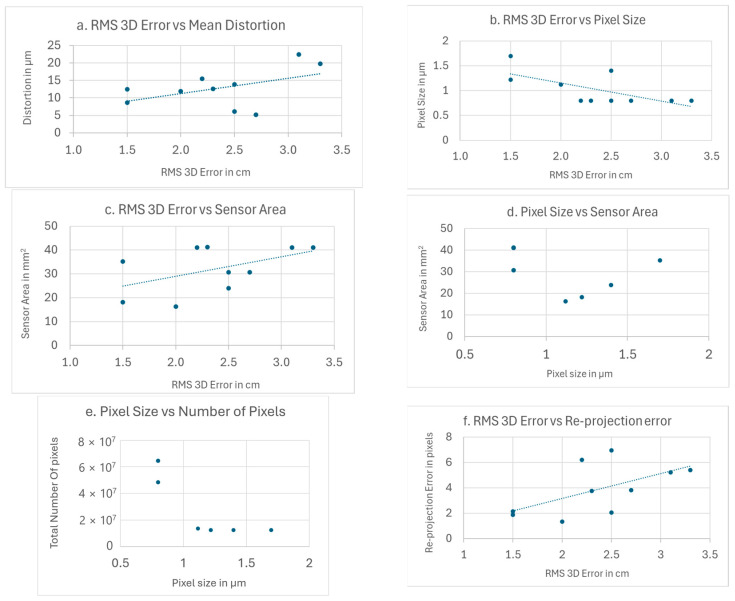
Scatter Charts for All Cameras with the trendline calculated by linear regression. (**a**): RMS 3D Error and Mean Distortion. (**b**) RMS 3D Error and Pixel Size. (**c**) RMS 3D Error and the Sensors Area. (**d**) Pixel Size and Sensor Area. (**e**) Pixel Size and Total Number of Pixels. (**f**) RMS 3D Error and Re-projection Error.

Figure 11 shows scatter charts of selected parameter combinations under consideration. In addition, a linear regression is performed and the trend line that best fits the point data is plotted to determine whether the relationship between them is linear. These charts can be used to examine how the accuracy of the photogrammetric methods is related to the technical characteristics of the cameras, e.g., the pixel size, sensor area, and total number of pixels. To determine whether the re-projection error can be an indicator of the accuracy of the results and to find out how the distortion is related to the accuracy. The cases examined are the RMS 3D error in relation to mean distortion in Figure 11a, RMS 3D error with pixel size in Figure 11b, RMS 3D error with sensor area in Figure 11c, pixel size and sensor area in Figure 11d, and pixel size and total number of pixels in Figure 11e. Figure 11f shows the RMS 3D error and the re-projection error.

## 4. Discussion of the Results

### 4.1. Distortions

For the camera calibration, the re-projection error in Table 2 ranges from 1.332 mm to 6.960 mm, with the results showing a range of 5.628 mm. The calibration results in Table 3 show that the calibrated value of the focal lengths deviates between 2% and 12% from the nominal focal lengths specified by the manufacturers. The aspect ratio varies from 0.998 to 1.003 and is therefore close to the central value of 1.000.

An examination of the distortions in Table 5 shows that they have a symmetrical form, with the distortion increasing from the center, which is zero, towards the edges, especially in the corners of the image, where it assumes the largest values. The symmetry is not absolute, indicating a transversal distortion. In some cases, such as with the Cam1, Cam2, and Cam9 cameras, there is a circular alteration in the colors, which shows a strong and undulating distortion variation.

It is worth mentioning the comparison of Cam1 with Cam2 and that of Cam3 with Cam7. These are the same models but different devices. In these cases, the distortion patterns show strong similarities, forming a signature that characterizes the camera of the respective smartphone model. Although the distortions have the same structure, they are not identical. The mean distortions are close, 22.45 μm against 19.78 μm for cameras Cam1 and Cam2 and 7.62 μm against 6.55 μm for cameras Cam3 and Cam7. In the distribution of distortions diagram in Figure 3, Cam1 and Cam2 have the same form but a clear shift. A similar situation appears in cameras 3 and 7. These changes may be due to the camera or to slight differences in the conditions under which the experiments are performed, which may slightly alter the results. The evaluation checkerboard may have the same dimensions in all experiments and the photographing positions may be predetermined, but small differences may occur. In any case, the observed differences in the experimental results are noteworthy and should be emphasized.

There appears to be a shift in the symmetry of the center of the Cam3, Cam7, and Cam8 cameras. One possible explanation is the built-in automatic image stabilization that some high-end smartphones have. This feature relies on the movement of the sensor or lens to compensate for the device’s movement when taking a photo so that it is not blurred. The result is visually better but leads to problems with variable distortion and unstable internal camera geometry. The absence of these side effects is a prerequisite for reliable images that can be used in photogrammetry. If these characteristics are uncontrollable, certain smartphone models should not be used. With smartphones, the various settings and functions that can be activated must be considered. However, this is not always easy to perceive, as the priorities with this technology are ease of use and automation.

In most cases, Table 8 shows a clear improvement in the undistortion process. Indeed, comparing the images of the first column to those of the second column of Table 8, there is always an improvement, particularly in the images’ central surface. This is confirmed in the third column of the table, which shows the differences in accuracy. In almost all cases, the green and blue colors, which show an improvement, dominate, while the orange and red colors, which indicate a decrease in accuracy, accumulate at the edges of the images. Yellow and cyan colors correspond to small changes and can be considered unchanged.

In Table 9 with the percentage changes of the ‘Rect’ indicator, it is observed that in most cases, there is a significant improvement in the photogrammetric rectification, ranging from +37.1% to +78.8%. In practice, this means that if, for example, in the first case, the undistorted image is used for photogrammetric rectification instead of the distorted original image, an overall improvement in geometric accuracy of 37.1% is achieved. In this way, it is possible to evaluate the quality of the distortion coefficients estimated by the calibration procedure. In one case, that of camera Cam6, there is a quantitative deterioration of the results by −0.6%. Even in this case, there is an improvement in the central part of the image and a deterioration in the edges. This is not a problem since, in general, using the photos’ edges in photogrammetry procedures is avoided.

### 4.2. Accuracy in Calculating Coordinates

According to Table 13, the mean RMSE for distorted image coordinates in the X and Y axes is between 1.3 cm and 1.4 cm, with a maximum estimated error of 2.2 cm, for a probability of 95.44%. In the scenario with undistorted image coordinates, the mean RMSE is 0.7 cm, and the maximum estimated error is 1.8 cm.

In the Z axis, when using the distorted image coordinates, the mean RMSE in the coordinate calculation is 1.6 cm, and the maximum predicted error is 2.8 cm. In the case where the undistorted image coordinates are used, the mean RMSE is 1.1 cm, and the maximum estimated error is 2.1 cm.

The mean RMSE in the calculation of the 2D coordinates, when the distorted image coordinates are used, is 2.0 cm, while when the undistorted ones are used, it is 1.1 cm, presenting an improvement of 45%. The maximum estimated error is 3.0 cm for the distorted and 2.2 cm for the undistorted. These errors correspond to creating scales of 1:150 and 1:110 for map products, respectively. However, if the camera Cam6, which achieved the best accuracy results, is used, the 1.3 cm RMSE of the distorted image coordinates corresponds to creating a scale of 1:130 for the map products, and the 0.6 cm RMSE, when the undistorted ones are used, corresponds to a scale of 1:60.

In the case of 3D coordinates, the mean RMSE is 1.6 cm when using the distorted image coordinates and 1.1 cm when using the undistorted ones, presenting an improvement of 19%. The maximum estimated error is 2.8 cm for the distorted ones and 2.1 cm for the undistorted ones.

The photographic distance, the number and distribution of control points and the use of commercial software using different and complex data analysis methods make it unreliable to compare the accuracy of the coordinate calculation between different scientific works. In this work, purely photogrammetric and topographic methods were used, which allow reliable conclusions to be drawn about the accuracy of the results.

### 4.3. Correlation Analysis

In the graph of Figure 10, the normalized mean values of the RMS 3D error and the mean rectification error are presented along with the normalized mean values of distortion, re-projection error, pixel size, sensor area, and total pixels of the imaging sensor.

Cameras Cam6 and Cam8 have the most accurate results in calculating actual coordinates, using distorted and undistorted coordinates. Both cameras have large pixel sizes, small numbers of total pixels, small re-projection errors, and middle mean distortion. Cam6 has a small sensor area and a small mean rectification error on distorted and undistorted image coordinates. However, Cam8 has a large sensor area and a large mean rectification error when distorted coordinates are employed. The mean rectification error is greatly improved when undistorted image coordinates are used. Cameras Cam5, Cam6, and Cam7 have an inferior performance in calculating the actual coordinates.

Cam1, Cam2, and Cam3 have the worst results in the accuracy of the coordinate calculation and show the smallest accuracy improvement when undistorted image coordinates are used. These sensors’ technical characteristics include a small pixel size, the largest sensor surface, a large number of pixels, moderate to high re-projection errors, large mean distortion, and variable rectification errors.

Cameras Cam9 and Cam10 have similar technical characteristics to those of Cam1, Cam2, and Cam3; however, they have better results in the accuracy of coordinate calculation, with an obvious improvement difference when using undistorted image coordinates.

Examining Figure 11a, in case distorted image coordinates are used, the RMSE in calculating 3D actual coordinates increases proportionally to the mean distortion. It is evident that there is not a perfect fit of the data to the linear model; however, the trend is given. The conclusion is expected, as the distorted coordinates introduce errors into the mathematical model of the collinearity equations used by the bundle adjustment method.

The RMS 3D error in Figure 11b,c indicates it is inversely proportional to the pixel size and proportional to the sensor area. These relationships can be interpreted based on the fact that the larger pixel size and larger sensor surface lead to greater radial distances where the actual points are displayed on the image. Increased radial distortion leads to larger errors in calculating actual coordinates.

Figure 11d,e show the relationship between the sensors’ technical characteristics. In Figure 11f, two groups of combinations can be distinguished. In one group, the sensor area increases proportionally to the pixel size that ranges from 1.2 to 1.7 μm, while in the second group, there is a constant smaller pixel size of 0.8 μm, corresponding to relatively large imaging sensor areas. Similarly, in Figure 11e, two groups are clearly distinguished. In the group with the largest pixel, the total number of pixels remains constant, but the pixel size increases. The size of the pixels remains constant in the second group and their total number changes using more pixels. What can be seen from the diagrams is the manufacturer’s strategy for making the sensors. On the one hand, there is a combination of small-sized pixels, with a large number and large sensor dimensions. On the other hand, increasing the sensor’s dimensions is attempted by using a larger pixel size.

As shown in Figure 11f, the 3D RMS error increases with the re-projection error. Therefore, the magnitude of the re-projection error can be an indication of how accurate the photogrammetric results are.

## 5. Conclusions

Every smartphone model has its distortion pattern, characterized by tangential distortion and undulating variation.

In most cases, there is a significant improvement in image undistortion, reaching up to 78.8%.

Cameras of the same smartphone model but different devices show strong but not identical similarities in distortions and accuracy in the photogrammetric calculation of actual coordinates.

Considering the accuracy test results from all cameras, at a shooting distance of 11 m, the mean RMSE achieved in the calculation of 2D actual coordinates is 2.0 cm. When distortions due to the lenses are considered, the RMSE improves to 1.1 cm. For a 95.44% probability, the maximum estimated error is 3.0 cm, while if distortions are considered, it is reduced to 2.2 cm. The mean RMSE in 3D coordinates is 2.6 cm when using distorted image coordinates and 1.5 cm when using undistorted image coordinates.

Cameras with the largest pixel size and a smaller image sensor area have better accuracy results.

The image stabilization function on smartphones must be considered when used in photogrammetry.

The re-projection error, outputted from the OpenCV calibration procedure, is an indication of the accuracy in calculating actual coordinates.

The conclusion is that, despite its limitations, this technology, if best practices are adhered to, can yield satisfactory results when high accuracy is not required. In any case, the camera must undergo calibration procedures to validate or improve performance.

It would have been desirable if the most modern smartphones currently on the market had taken part in the study, but this was not feasible. However, the heterogeneity of technical features of the more affordable devices that were available and used provided an overall picture of the capabilities of this technology. In the future, it would be interesting to compare generations of sensors and identify changes in performance.

## Figures and Tables

**Table 1:** Smartphone Cameras Technical Specifications.

Camera Code	Smartphone Model	Nominal Focal Length (mm)	Sensor Resolution (pixels)	Pixel Size (μm)	Physical Dimensions of the Sensor (mm)
Cam1	Samsung Galaxy A52s	5	9248 × 6936	0.80	7.398 × 5.549
Cam2	Samsung Galaxy A52s	5	9248 × 6936	0.80	7.398 × 5.549
Cam3	Xiaomi Redmi Note 7	5	8000 × 6000	0.80	6.400 × 4.800
Cam4	Xiaomi Redmi 9	4	4160 × 3120	1.12	3.915 × 3.908
Cam5	iPhone 12	4	4032 × 3024	1.40	5.645 × 4.234
Cam6	iPhone 8 Plus	4	4032 × 3024	1.22	4.919 × 3.689
Cam7	Xiaomi Redmi Note 7	5	8000 × 6000	0.80	6.400 × 4.800
Cam8	iPhone 13 Wide	4	4032 × 3024	1.70	6.854 × 5.141
Cam9	Samsung Galaxy A71	5	9248 × 6936	0.80	7.398 × 5.549
Cam10	Xiaomi Redmi Note 9 Pro	5	9280 × 6944	0.80	7.424 × 5.555

**Table 2:** The re-projection error when calibrating cameras with OpenCV.

Camera	Re-Projection Error (mm)
Cam1	5.206
Cam2	5.390
Cam3	6.960
Cam4	1.332
Cam5	2.049
Cam6	1.866
Cam7	3.805
Cam8	2.131
Cam9	6.214
Cam10	3.765

**Table 3:** The Intrinsic Matrices for all cameras.

Camera	*f_x_* (pixels)	*f_y_* (pixels)	*c_x_* (pixels)	*c_y_* (pixels)	*AspectRatio* (*f_y_*/*f_x_*)	Estimated *c* (mm)
Cam1	6844.1	6830.6	4659.0	3485.1	0.998	5.475
Cam2	6871.4	6864.9	4619.5	3471.6	0.999	5.497
Cam3	6030.1	6025.5	4014.5	2956.6	0.999	4.824
Cam4	3495.4	3489.5	2095.0	1568.7	0.998	3.915
Cam5	3080.3	3085.5	2005.7	1505.2	1.002	4.312
Cam6	3347.9	3358.9	1994.2	1477.5	1.003	4.085
Cam7	6072.4	6082.2	3979.7	2954.0	1.002	4.858
Cam8	3128.4	3130.3	2028.6	1509.4	1.001	5.318
Cam9	6790.6	6793.8	4685.2	3509.4	1.000	5.433
Cam10	6980.9	6995.8	4664.4	3459.7	1.002	5.585

**Table 4:** The Distortion Coefficients Parameters for all cameras.

Camera	*k*_1_	*k*_2_	*k*_3_	*p*_1_	*p*_2_
Cam1	0.11334916	−0.35944055	0.32451562	0.00071273	−0.00043841
Cam2	0.09919886	−0.30325452	0.25820729	0.00032525	−0.00052421
Cam3	0.02874193	−0.08315243	0.02510906	−0.00216489	0.00029807
Cam4	0.10052372	−0.37294230	0.42898550	0.00137681	−0.00038364
Cam5	0. 10977028	−0.25010756	0.00040956	−0.00104321	0.17731282
Cam6	0.12008488	−0.53374705	0.70438302	0.00081659	−0.00143819
Cam7	0.02610525	−0.05473527	0.00663625	−0.00089408	−0.00026689
Cam8	0.01878580	0.03268274	−0.16269373	0.00037758	−0.00227720
Cam9	0.10503463	−0.33778408	0.26182562	−0.00036476	0.00020589
Cam10	0.06732571	−0.25357517	0.25779797	−0.00007694	−0.00038661

**Table 5:** Evaluation images displaying distortions per pixel.

Camera	Distortions	Mean Distortion (st.Dev) in Pixels	Mean Distortion (st.Dev) in μm
Cam1	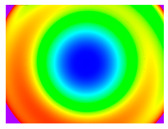	28.06 (13.21)	22.45 (10.56)
Cam2	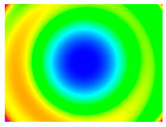	24.73 (11.50)	19.78 (9.20)
Cam3	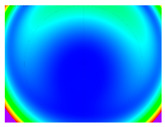	7.62 (8.12)	6.10 (6.50)
Cam4	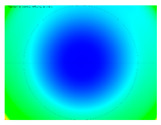	10.67 (5.95)	11.95 (6.67)
Cam5	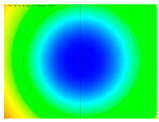	17.33 (10.24)	13.86 (8.19)
Cam6	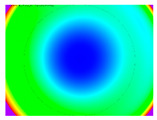	10.19 (6.16)	12.43 (7.51)
Cam7	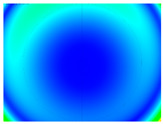	6.55 (4.22)	5.24 (3.38)
Cam8	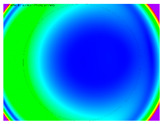	5.12 (5.60)	8.71 (9.52)
Cam9	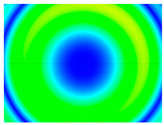	19.40 (10.21)	15.52 (8.16)
Cam10	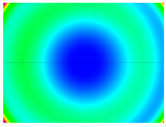	12.56 (5.81)	10.05 (4.65)

**Table 6:** Max Rectification Error in the Evaluation Images.

Camera	Max Rectification Error (mm)	
	Distorted Image	Undistorted Image
Cam1	0.879	0.593
Cam2	0.995	0.788
Cam3	1.381	1.346
Cam4	0.422	0.348
Cam5	0.277	0.174
Cam6	0.401	0.533
Cam7	0.826	0.421
Cam8	1.977	1.007
Cam9	1.728	1.156
Cam10	0.796	0.669

**Table 7:** Color Gradients for the Undistortion Performance.

Error Differences (mm)	Color Gradients
error diff. > 0.3	
0.2 < error diff. < 0.3	
0.1 < error diff. < 0.2	
0 < error diff. < 0.1	
−0.1 < error diff. < 0	
−0.2 < error diff. < −0.1	
error diff. < −0.2	

**Table 8:** Evaluation Images Displaying the Rectification Errors and the Undistortion performance.

Camera	Rectification Errors in the Distorted Image	Rectification Errors in the Undistorted Image	Undistortion Performance
Cam1	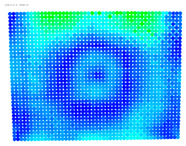	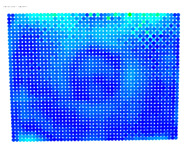	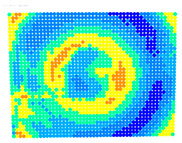
Cam2	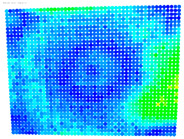	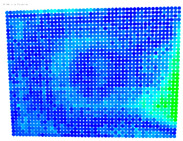	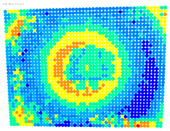
Cam3	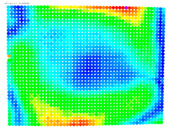	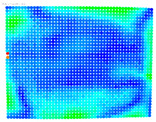	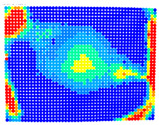
Cam4	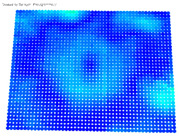	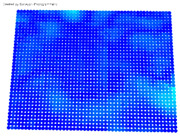	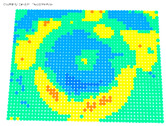
Cam5	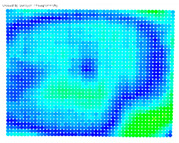	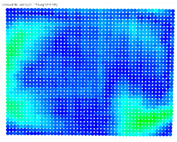	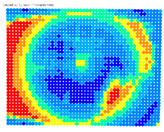
Cam6	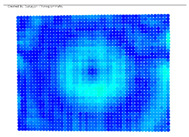	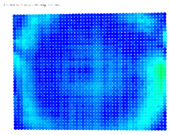	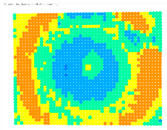
Cam7	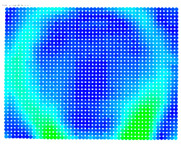	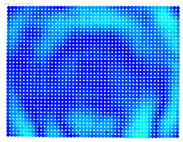	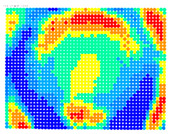
Cam8	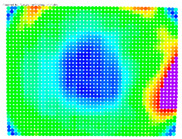	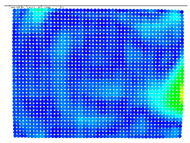	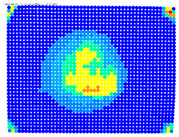
Cam9	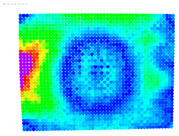	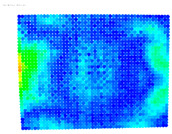	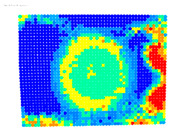
Cam10	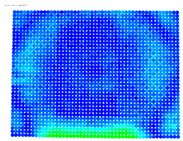	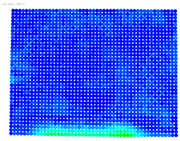	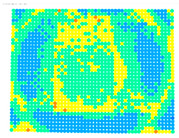

**Table 9:** Evaluation results for the undistortion performance.

Camera	Mean Rectification Error (St.Dev) in mm		the ‘Rect’ Indicator
	Distorted Image	Undistorted Image	
Cam1	0.208 (0.129)	0.114 (0.081)	+45.2%
Cam2	0.250 (0.179)	0.141 (0.116)	+43.6%
Cam3	0.509 (0.293)	0.224 (0.147)	+55.8%
Cam4	0.115 (0.074)	0.067 (0.046)	+41.0%
Cam5	0.277 (0.151)	0.174 (0.131)	+37.1%
Cam6	0.139 (0.088)	0.140 (0.095)	−0.6%
Cam7	0.206 (0.156)	0.124 (0.082)	+39.8%
Cam8	0.591 (0.339)	0.125 (0.120)	+78.8%
Cam9	0.369 (0.298)	0.198 (0.161)	+46.2%
Cam10	0.125 (0.113)	0.076 (0.082)	+39.1%

**Table 10:** The Bundle Adjustment Solutions.

Camera	Observations	Control Points	Check Points	Distorted		Undistorted	
				Sigma	Degrees of Freedom	Sigma	Degrees of Freedom
Cam1	62	10	11	6.406	70	3.945	65
Cam2	53	10	10	11.472	55	9.345	50
Cam3	58	10	11	4.357	62	2.380	57
Cam4	54	10	11	2.221	54	1.290	49
Cam5	62	10	11	2.529	70	1.488	65
Cam6	62	10	11	1.411	70	0.788	65
Cam7	62	10	11	3.779	70	1.405	65
Cam8	60	10	11	1.658	66	1.056	61
Cam9	52	10	10	6.830	53	2.270	48
Cam10	62	10	11	4.421	70	2.088	65

**Table 11:** Checkpoints RMSE in the X, Y, and Z axis using Bundle Adjustment.

Camera	Distorted (cm)			Undistorted (cm)		
	X	Y	Z	X	Y	Z
Cam1	2.0	2.2	1.5	1.1	1.1	1.4
Cam2	1.7	1.7	2.7	1.9	1.7	1.7
Cam3	1.3	1.3	2.2	0.9	0.5	2.0
Cam4	1.3	1.2	1.2	0.6	0.8	0.8
Cam5	1.8	1.2	1.5	0.7	0.6	1.3
Cam6	1.1	0.7	1.0	0.5	0.3	0.9
Cam7	1.2	1.4	2.3	0.3	0.5	1.0
Cam8	1.0	0.9	1.0	0.4	0.6	0.6
Cam9	1.5	1.5	1.1	0.4	1.7	0.5
Cam10	1.3	1.2	1.7	0.4	0.5	0.8

**Table 12:** Checkpoints RMSE in the XY level and 3D using Bundle Adjustment.

Camera	XY (cm)		3D (cm)	
	Distorted	Undistorted	Distorted	Undistorted
Cam1	3.0	1.6	3.3	2.1
Cam2	2.4	2.5	3.6	3.0
Cam3	1.9	1.0	2.9	2.2
Cam4	1.8	1.0	2.2	1.3
Cam5	2.2	1.0	2.7	1.6
Cam6	1.3	0.6	1.6	1.0
Cam7	1.8	0.7	2.9	1.2
Cam8	1.3	0.7	1.6	0.9
Cam9	2.3	0.7	2.6	0.8
Cam10	1.7	0.7	2.4	1.1

**Table 13:** Mean RMSEs and Maximum Estimated Errors for X, Y, Z, XY, and 3D.

Parameter	Distorted/ Undistorted	Mean RMSE Value (cm)	Max Estimated Error (cm) 95.44% Probability
X	Distorted	1.4	2.1
	Undistorted	0.7	1.7
Y	Distorted	1.3	2.2
	Undistorted	0.8	1.8
Z	Distorted	1.6	2.8
	Undistorted	1.1	2.1
XY	Distorted	2.0	3.0
	Undistorted	1.1	2.2
3D	Distorted	2.6	3.9
	Undistorted	1.5	2.9

## Data Availability

Data are contained within the article.
